# Leri-Weill Dyschondrosteosis Syndrome: Analysis via 3DCT Scan

**DOI:** 10.3390/medicines6020060

**Published:** 2019-05-29

**Authors:** Ali Al Kaissi, Mohammad Shboul, Vladimir Kenis, Franz Grill, Rudolf Ganger, Susanne Gerit Kircher

**Affiliations:** 1 Ludwig Boltzmann Institute of Osteology, at the Hanusch Hospital of WGKK and, AUVA Trauma Centre Meidling, First Medical Department, Hanusch Hospital, Vienna 1140, Austria; 2 Paediatric department, Orthopaedic Hospital of Speising, Vienna 1130, Austria; grill.franz@gmx.net (F.G.); rudolf.ganger@oss.at (R.G.); 3 Department of Medical Laboratory Sciences, Jordan University of Science and Technology, Irbid 22110, Jordan; mohammad.shboul@reversade.com; 4 Department of Foot and Ankle Surgery, Neuroorthopaedics and Systemic Disorders, Pediatric Orthopedic Institute n.a. H. Turner, Parkovaya str., 64–68, Pushkin, Saint Petersburg, Russia; kenis@mail.ru; 5 Department of Medical Chemistry, Medical University of Vienna, Vienna 1090, Austria; susanne.kircher@meduniwien.ac.at

**Keywords:** Leri-Weil dyschondrosteosis, tomography, craniosynostosis, deficient ribs number, ischial dysplasia, coxa valga, *SHOX* gene

## Abstract

**Background:** Leri-Weill dyschondrosteosis (LWD) is a pseudoautosomal form of skeletal dysplasia, characterized by abnormal craniofacial phenotype, short stature, and mesomelia of the upper and lower limbs. **Methods:** We describe two female patients with LWD. Their prime clinical complaints were severe bouts of migraine and antalgic gait. **Results:** Interestingly, via a 3D reconstruction CT scan we encountered several major anomalies. Notable features of craniosynostosis through premature fusion of the squamosal sutures and partial closure of the coronal sutures were the reason behind the development of abnormal craniofacial contour. A 3D reconstruction CT scan showed apparent bulging of the clavarium through the partially synostosed coronal and totally synostosed squamosal sutures. Additional deformities include deficient number of ribs (10 ribs on both sides), defective ossification of the ischium and dysplasia of the iliac-ischial junction, and coxa valga have been noted. **Conclusions:** The constellation of observed deformities can be considered as a novel features associated with LWD.

## 1. Introduction

Leri-Weill dyschondrosteosis (LWD; OMIM 127300) is a pseudoautosomal form of skeletal dysplasia characterized by mesomelic limb shortening, short and broad forearms due to unusual curvature of the radius, and Madelung´s deformity of the wrist, which is usually clinically apparent [1,2,3]. Genu valgum is also common [4]. Stature is reduced to between 137 and 152 cm. Radiographs reveal bowing of the radius and ulna, whose distal ends form a “V-shaped” configuration between which the carpals are wedged. The ulnar styloid is usually posteriorly dislocated. In the lower limbs, the tibia and fibula are short (with the latter being more severely affected) [5,6,7]. 

LWD is predominantly caused by haploinsufficiency of the short stature homeobox-containing gene (*SHOX*; OMIM 312865), which accounts for approximately 80% of reported cases. The genetic defect remains unknown in around 30% of LWD cases [8]. The *SHOX* gene is located in the pseudoautosomal region 1 (PAR1) of the sex chromosomes (Xp22.33, Yp11.32). Therefore, both females and males have two functional copies of *SHOX* and show a pseudoautosomal inheritance pattern [9]. Around the gene, there are at least seven evolutionarily conserved non-coding DNA elements (CNEs) that act as enhancers [10,11,12,13]. 

*SHOX* encodes a transcription factor implicated in human growth and skeletal development [14] and plays a crucial role in the chondrocyte function of the growth plate as a regulator of cellular proliferation and differentiation [15,16]. *SHOX* haploinsufficiency results from heterozygous point mutations in the coding exons and/or copy number variation (CNV) in *SHOX* and/or the upstream and downstream enhancer regions of *SHOX*. In addition to LWD, heterozygous alterations have also been reported in individuals with different short stature syndromes, such as idiopathic short stature (ISS; OMIM 300582) without skeletal phenotypes, which accounts for 2%–16% of children [2,11,17,18,19], and Turner syndrome (OMIM 127300) [14,19,20]. Moreover, the loss of both *SHOX* alleles due to homozygous or compound heterozygous leads to the complete lack of *SHOX* and causes a severe and extreme phenotype of osteodysplasia called Langer mesomelic dysplasia (LMD; OMIM 249700) [2,21,22]. Odontoid hypoplasia has been reported in a long list of skeletal dysplastic disorders [23]. 

The purpose of this paper is to further understand the etiology behind the frequent severe bouts of migraine and the antalgic gait. 

## 2. Materials and Methods

### 2.1. Participants and Ethics

The study protocol was approved and obtained from the Hospital Ethical Committee (Ethics Committee of the Turner Scientific Research Institute (January 5, 2016), No.3/2016, Saint-Petersburg). In addition, informed consent was obtained from the patient’s guardians. The records of two girls with the phenotype and genotype of LWS were studied at the osteogenetic department of the Orthopaedic Hospital of Speising, Vienna, Austria.

### 2.2. Clinical Examination

A 17-year-old female child was referred because of waddling gait and pelvic pain (antalgic gait). In her early childhood, the complaint of severe bouts of migraine was a constant reason to seek medical advice. She was a product of non-consanguineous parents. At birth, her length and weight were around the 10th percentile, and her OFC was around the 50th percentile. Her subsequent course of development has been within the normal limits. Clinical examination showed short stature (−5 SD) associated with a seemingly large and abnormal head contour (75th percentile) (the cranial deformity was extremely unusual, there was extensive deformity of the cranial vault extended from the borders of the cranial sutures involving the posterior aspect of the cranium causing severe bulging of the temporal bones). 

The upper limbs appeared short and the mobility of the arms were decreased, where this was more apparent in the forearms (problems in pronation and supination) and was associated with weakness of the muscles in both arms and forearms. We observed limitations of the sternal movement and these were associated with impairment of the sternal expansion. The patient had a history of gradual pain around the hip (ischial tuberosity after activities in sports or long-distance walking). Tenderness on palpation of the ischial tuberosity has been elicited. Neurological examination was normal. Hearing and vision were normal, but her schooling achievement was borderline. Examination of hair, nails, and skin were normal, but enamel dysplasia and persistence of the primary teeth was a notable feature associated with problems in mastication. In order to exclude papilledema as a possible reason behind her migraine, a funduscopic examination was performed, and no signs of increased intracranial pressure were elicited. 

The second patient was a 15-year-old girl with typical clinical phenotypic characterizations. In addition, she manifested abnormal bowing of the legs (genu varum). Joint examination showed bilateral coxa valga associated with painful limitation of external and internal rotation of both hip joints. A waddling gait associated with painful knee joints were the most prominent deformities. The neurological examination was normal. Hearing and vision were normal. However, her intelligence was borderline. Examination of hair, teeth, nails, and skin were normal.

### 2.3. Laboratory Measurements

Laboratory investigations of serum proteins, calcium, phosphorus, alkaline phosphatase levels, urinary amino acids, and mucopolysaccharides were undertaken. Erythrocyte sedimentation rate (ESR), HLA-B27, renal ultrasound, and chromosomal karyotyping were also carried out.

## 3. Results

**Figure 1:**
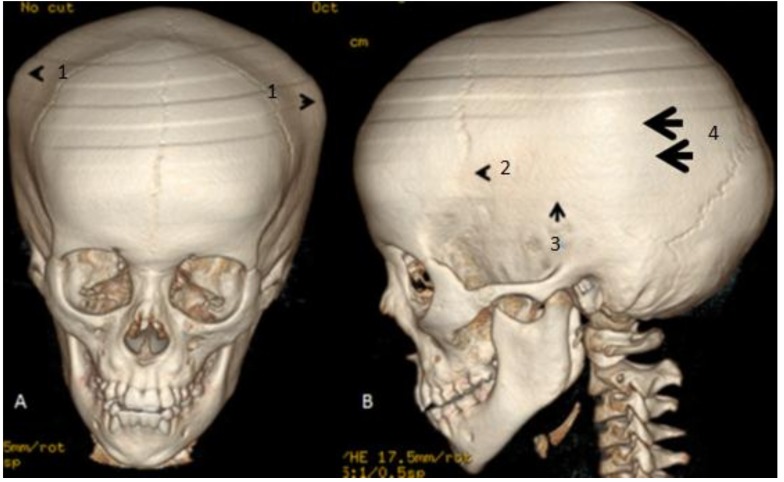
(**A**,**B**) 3D reconstruction CT scan of the cranium (anterior and lateral views) in the 17-year-old girl showed marked bulging of the frontal area associated with overlapping of the posterior cranial vault because of extreme bulging of the temporal bones (hat-like appearance in connection with extreme bulging of the skull bones over the temporal areas (arrowheads1). Partial closure of the coronal sutures (arrowhead 2) associated with total closure of the squamosal sutures (arrow 3) resulted in the development of bony bulging over the synostosed squamosal sutures. The bony bulge extended over the squamosal suture arches posteriorly from the pterion and connected the temporal squama with the inferior border of the parietal bone (hat-like appearance in the anterior view) (arrowheads 1) and (arrows 4) in the lateral view 4. Note the persistence of the metopic, sagittal, and lambdoid sutures (Figure 1A,B).

We referred to a 3DCT scan to further analyze the malformation complex in this patient. We used a GE light speed 16 CT Scanner (300mA, 120kv). Rotation 0.80 s/HE 13.8 mm/rot 0.6 mm 1.375:1/0.6 sp (reconstruction: volume rendering). 3D reconstruction CT scan of the cranium (anterior and lateral views) in the 17-year-old girl showed marked bulging of the frontal area associated with overlapping of the posterior cranial vault because of extreme bulging of the temporal bones (hat-like appearance in connection with extreme bulging of the skull bones over the temporal areas (arrowheads)). Partial closure of the coronal sutures (arrowhead) associated with total closure of the squamosal sutures (arrow) resulted in the development of bony bulging over the synostosed squamosal sutures. The bony bulge extended over the squamosal suture arches posteriorly from the pterion and connected the temporal squama with the inferior border of the parietal bone (hat-like appearance in the anterior view) (arrowheads) and arrows in the lateral view. Note the persistence of the metopic, sagittal, and lambdoid sutures (Figure 1A,B). 

**Figure 2:**
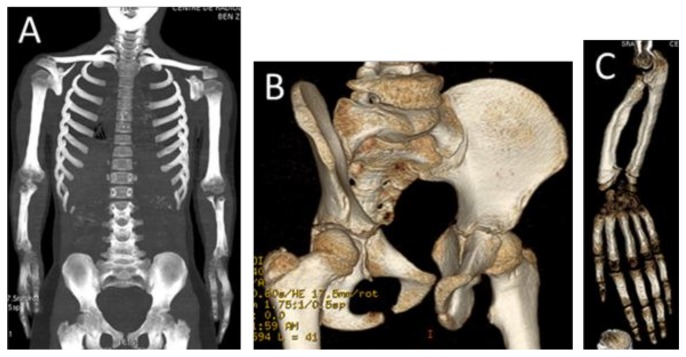
Reconstruction CT scan of the whole skeleton showed rhizomelia, metaphyseal widening of the humeri and a deficient number of ribs (10 instead of 12) (**A**). Pelvic bones showed coxa valga and iliac–ischial dysplasia due to defective ossification of the ischial rami (**B**). The forearms showed Madelung´s deformity (short radius and radial dislocation with lateral and posterior bowing, a triangular epiphysis due to absence of the growth plate medially, and posterior dislocation the lower end of the ulna), the carpal bones were wedged between the distal ends of the radius and protruding ulna (**C**).

**Figure 3:**
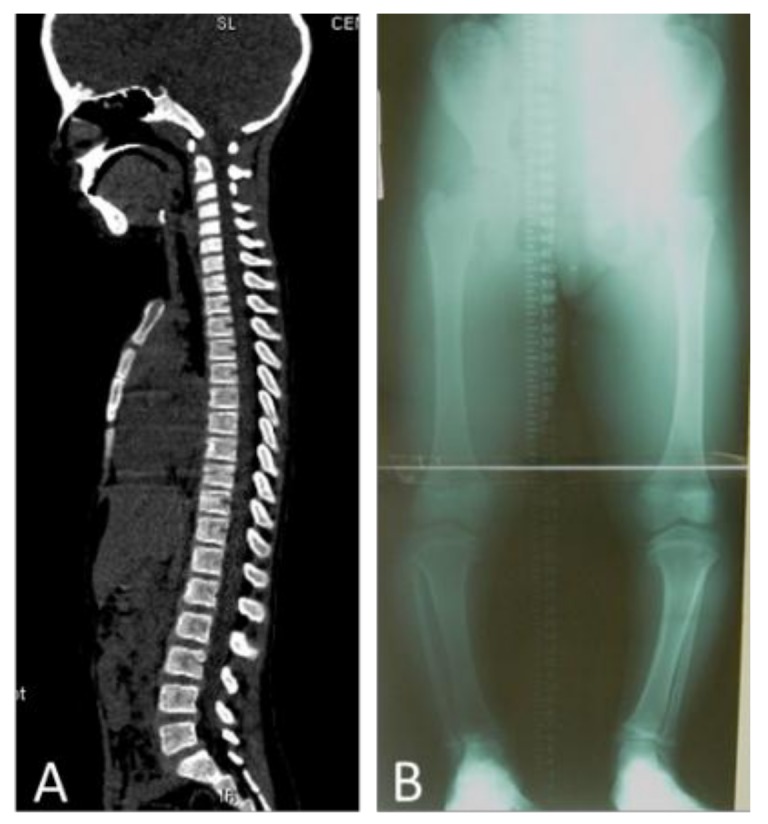
(**A**) 3DCT scan of the vertebral column (mid-sagittal) of a 17-year-old girl showed odontoid hypoplasia and square-shaped vertebral bodies with marked anterior end-plate dysplasia of the T9-11. (**B**) AP radiograph of the lower limbs (telemetric) in a 15-year-old girl showed leg shortening relative to the thigh, and metaphyseal widening of the inferior femora and the superior tibiae effectively causing the development of genu varum.

A reconstruction CT scan of the whole skeleton showed short limbs, metaphyseal widening of the humeri, and a deficient number of ribs (10 instead of 12) (Figure 2A). Pelvic bones showed coxa valga and iliac–ischial dysplasia due to defective ossification of the ischial rami (Figure 2B). The forearms showed Madelung´s deformity (short radius and radial dislocation with lateral and posterior bowing, triangular epiphysis due to the absence of the growth plate medially, and posterior dislocation the lower end of the ulna), and the carpal bones were wedged between the distal ends of the radius and protruding ulna (Figure 2C). The 3DCT scan of the vertebral column (mid-sagittal) of the 17-year-old girl showed odontoid hypoplasia and square-shaped vertebral bodies with marked anterior end-plate dysplasia of the T9-11 (Figure 3A). (Figure 3B) AP radiograph of the lower limbs (telemetric) in the 15-year-old girl showed leg shortening relative to the thigh (mesomelia of the lower limbs), and metaphyseal widening of the inferior femora and the superior tibiae causing effectively the development of genu varum (Figure 3A,B).

Laboratory investigations showed normal white and red blood cell and platelet counts, and normal serum calcium, phosphorus, and alkaline phosphatase levels. Further blood analyses showed normal serum total protein, and the albumin-globulin ratios were within the normal range. Urine amino acids and mucopolysaccharides were normal. The erythrocyte sedimentation rate (ESR), HLA-B27, and renal ultrasound were also normal. Chromosomal analysis showed a normal karyotype. Both patients underwent genetic tests and the results demonstrated common heterozygous deletion around the *SHOX* gene.

## 4. Discussion

Dyschondrosteosis is the most common of a number of varieties of mesomelic dysplasia affecting only the middle segment of the extremities. Leri and Weil [1] first identified this type and gave it the name dyschondrosteosis. Dyschondrosteosis is of common occurrence in our department, and in a vast majority of cases are presented with minimal disability; therefore, many remain undiagnosed.

It is characterized by moderate shortness of stature due to short tibia and fibula, and short forearms, often with a wrist deformity, similar to Madelung’s deformity. Langer et al. [7], Anton et al. [21], and Dannenberg et al. [22] identified many cases that were said to be Madelung’s deformity but the presence of short stature and a hereditary influence suggested that they were instances of dyschondrosteosis.

Maroteaux and Lamy [5], Langer et al. [7], and Dawe, et al. [23] have added further families and stress the clinical variation in the condition. Inheritance was autosomal dominant with only 50% penetrance. 

Belin et al. [24] and Shears et al. [25] demonstrated common deletions around the *SHOX* gene. Point mutations leading to likely haploinsufficiency were also demonstrated. Shears et al. [25] showed homozygous deletion of the *SHOX* gene in a fetus with Langer mesomelia. Belin et al. [24] showed that an XO fetus with features of Langer mesomelia was hemizygous for a deletion of the *SHOX* gene. Rao et al. [26] suggested that point mutations in the *SHOX* gene gave an idiopathic short stature; however, the family they reported was not described in great clinical detail and may in fact have manifested LWD. The point mutation in the Rao et al. [26] family was the same as that seen in the Belin et al. [24] family. Further point mutations were reported by Grigelioniene et al. [27]. Schiller et al. [28] studied 18 families with phenotypic features of Leri-Weill syndrome and found microscopic deletions involving the *SHOX* gene in 10 (59%) of the families. No point mutations were found in the other 8 families, suggesting possible genetic heterogeneity. Musebeck et al. [29] studied 35 cases with idiopathic short stature and found no evidence of *SHOX* deletions. 

Clement-Jones et al. [14] examined the expression pattern of *SHOX* and its homolog, *SHOX2* on 3q, and found that this supported its role in the phenotype of LWD and Turner syndromes.

Craniosynostosis is an early fusion of the skull sutures, which can be part of a long list of syndromic entities, and can occur as a sporadic occurrence, but nevertheless, craniosynostosis was not previously reported as an associated abnormality in LWD.

Thomas et al. [30] reported on 217 children with nonsyndromic sagittal synostosis followed for a mean of 86 months. The overall rate of raised ICP following sagittal synostosis surgery was 6.9%, found at an average of 51 months after initial surgery. Two types of surgery had different outcomes at this British institution: 1.6% (2 out of 128 patients) who underwent calvarial remodeling versus 14.6% (13 out of 89 patients) who underwent modified sagittal strip craniectomy developed raised ICP.

Hypoplasia of the ischia is an extremely rare congenital malformation, which has been reported in very few cases, and it has been known as a syndromic constituent in only a few congenital malformations syndromes, such as ischio-vertebral dysplasia, ischio-patellar hypoplasia, complex severe limb anomalies in postaxial acrofacial dysostosis, and can be observed in Treacher-Collins syndrome [31].

Cohen et al. [32] described a disorder, which is resembles Cleido-cranial dysplasia, but severe kyphoscoliosis with defective ossification of the ischial rami were the prime features, though there was preservation of the clavicles.

Ischio-spinal dysostosis is another clinical entity described by Nishimura et al. [33] in which hypoplasia of the ischial rami and multiple segmental anomalies of the spine were described as a recognizable pattern of malformations. 

It is mandatory to differentiate Leri-Weil dyschondrosteosis syndrome from other forms of mesomelic dysplasia. Nievergelt [34] described a syndrome of gross symmetrical deformities of the distal parts of the limbs associated with dislocation of the radial head, radial synostosis, tarsal synostosis, and talipes equinovarus; however, no ischial hypoplasia and craniosynostosis were reported.

Langer et al. [7] reported two cases as mesomelic dwarfism in which there was marked hypoplasia of the distal ulna and the proximal fibula, the dwarfism was severe, and the inheritance pattern was compatible with the autosomal recessive transmission. Similarly, neither ischial hypoplasia nor craniosynostosis were reported.

Mesomelic dysplasia is also a feature of Robinow syndrome [35] with a characteristic facial changes (fetal face syndrome), where radiologically, there is shortening of the radius and the ulna, hypoplasia of the distal part of the humerus, multiple rib abnormalities, multiple vertebral abnormalities and scoliosis, and lobster claw feet, and the inheritance is autosomal recessive.

## 5. Conclusions

In almost all the reported patients with Leri-Wiel dyschondrosteosis, neither craniosynostosis nor ischial hypoplasia have been described. Following the clinical examination of the cranium, and in order to confirm or rule out craniosynostosis, the radiological examination of choice was the 3D reconstruction CT scan, which contributed positively in delineating the skull bones phenotype and to further understand the reason behind the abnormal craniofacial contour and the associated bouts of migraine and borderline schooling achievement. The frequent bouts of migraine and the antalgic gait associated with progressive waddling gait are novel clinical presentations in patients with LWD. An abnormal craniofacial contour was a distinctive feature that was explained via a 3D reconstruction CT scan as in connection with early closure of the squamosal and partial closure of the coronals. Craniosynostosis is an unusual clinical feature in this syndromic entity. The father and a paternal female sibling of a 31-year-old had a history of migraines and she clinically manifested a similar craniofacial contour but with a moderate short stature.
